# Aerobic vaginitis is associated with carbonic anhydrase IX in cervical intraepithelial neoplasia

**DOI:** 10.1038/s41598-024-57427-x

**Published:** 2024-04-16

**Authors:** Švitrigailė Grincevičienė, Daiva Vaitkienė, Daiva Kanopienė, Rasa Vansevičiūtė Petkevičienė, Artūras Sukovas, Joana Celiešiūtė, Ernesta Ivanauskaitė Didžiokienė, Arvydas Čižauskas, Aida Laurinavičienė, Dovilė Stravinskienė, Jonas Grincevičius, Daumantas Matulis, Jurgita Matulienė

**Affiliations:** 1 Department of Biothermodynamics and Drug Design, Institute of Biotechnology, Life Sciences Center, Vilnius University, Sauletekio Av. 7, 10257 Vilnius, Lithuania; 2 Department of Obstetrics and Gynecology, Medical Academy, Lithuanian University of Health Sciences, Eiveniu St. 2, 50161 Kaunas, Lithuania; 3 Consultative Polyclinic Department, National Cancer Institute, Santariskiu St. 1, 08406 Vilnius, Lithuania; 4 Clinic of Obstetrics and Gynecology, Faculty of Medicine, Institute of Clinical Medicine, Vilnius University, M. K. Ciurlionio St. 21, 03101 Vilnius, Lithuania; 5 National Center of Pathology, Affiliate of Vilnius University Hospital Santaros Klinikos, P. Baublio St. 5, 08406 Vilnius, Lithuania; 6 Department of Pathological Anatomy, Medical Academy, Lithuanian University of Health Sciences, Eiveniu St. 2, 50161 Kaunas, Lithuania; 7 Department of Pathology, Forensic Medicine and Pharmacology, Faculty of Medicine, Institute of Biomedical Science, Vilnius University, M. K. Ciurlionio St. 21, 03101 Vilnius, Lithuania; 8 Department of Immunology and Cell Biology, Institute of Biotechnology, Life Sciences Center, Vilnius University, Sauletekio Av. 7, 10257 Vilnius, Lithuania; 9 Faculty of Medicine, Pharmacy and Pharmacology Center, Institute of Biomedical Science, Vilnius University, M. K. Ciurlionio St. 21, 03101 Vilnius, Lithuania

**Keywords:** CAIX enzyme, Carbonic anhydrase 9, Cervical intraepithelial neoplasia, Uterine cervix dysplasia, Aerobic vaginitis, Microbiota alteration, Cervical cancer, Microbial communities

## Abstract

The aim of this study was to analyze the association between vaginal microbiota, carbonic anhydrase IX (CAIX) and histological findings of cervical intraepithelial neoplasia (CIN). The study included 132 females, among them 66 were diagnosed with high-grade intraepithelial lesion (CIN2, CIN3, and cancer), 14 with low-grade disease, and 52 assigned to the control group. An interview focused on the behavior risk factors, together with vaginal fluid pH measurement, wet mount microscopy, detection of Chlamydia trachomatis, and Trichomonas vaginalis were performed. After colposcopy, high-grade abnormalities were detected via direct biopsies and treated with conization procedure. Conuses were immuno-stained with CAIX antibody. The histological findings were CIN1 (n = 14), and CIN2+ (included CIN2 (n = 10), CIN3 (n = 49), and cancer (n = 7; squamous cell carcinomas)). Prevalence of bacterial vaginosis (BV) was similar between the groups. Moderate or severe aerobic vaginitis (msAV) was diagnosed more often among CIN2+ (53.0%) than CIN1 (21.4%). Moderate or strong immunostaining of CAIX (msCAIX) was not detected among CIN1 cases. Thus, msAV was prevalent in CAIX non-stained group (p = 0.049) among CIN2 patients. Co-location of msAV and msCAIX was found in CIN3. Regression model revealed that msAV associated with high-grade cervical intraepithelial neoplasia independently from smoking and the number of partners.

## Introduction

Persistence of high-risk human papillomavirus (hr-HPV) infection highly increases the probability for the development of cervical intraepithelial neoplasia (CIN) and consequently cancer^1^. Surgical excision and pathologic evaluation of specimens for precancerous lesion or carcinomas is a golden standard for precise diagnosis. Novel treatment with prembrolizumab provides hope for severe ill people^2^. However, the World Health organization aims acceleration of rapid elimination of cervical cancer as the prioritized public health goal^3^. Keeping in mind that vaccination against hr-HPV infection covers only small part of hr-HPV variances^4^, the analysis and modification of risk factors for hr-HPV clearance is a very promising strategy.

Spontaneous clearance is possible, but the number of factors is associated with an elevated risk for hr-HPV genome integration into the host deoxyribonucleic acid (DNA) resulting in carcinogenesis^5^. Inflammation and oxidation stress aggravated by smoking, alteration of immunity, and Chlamydia trachomatis (*C. trachomatis*) infection contribute to oncogenesis in the uterine cervix^6^. Other detectable bacteria (such as *Mycoplasma* spp. and *Ureaplasma* spp.) are under research^7^.

Higher number of partners was also described as a factors for increased risk of hr-HPV acquisition, but cultural differences could interfere probability to develop CIN2 or more advance neoplasm^8^. However, these factors do not cause a disease. They counterplaying with genes of patients modifying hr-HPV and host interaction^9^.

Human microbiome study revealed that abnormal vaginal environment plays a key role in women’s urogenital pathology^10^. Furthermore, a meta-analysis showed an association of bacterial vaginosis (BV) and CIN^11^. In addition, increasing number of publications report the correlation of high-grade intraepithelial neoplasia (HSIL), that includes CIN2+, with aerobic vaginitis (AV) or mixed AV-BV flora^12,13^.

Following the first publication in 2002 by Donders et al.^14^, AV is an abnormal vaginal flora lacking lactobacilli, but abundant in aerobic bacteria, inflammation and parabasal cells. The presence of proinflammatory cytokines (interleukin (IL)-1 and IL-6) detected among women with AV supports the hypothesis of its role in hr-HPV-related pathogenesis^15^.

Carbonic anhydrase IX (CAIX) is an enzyme located on the eucaryotic cell membrane and is responsible for the production of bicarbonate and acid hydrogen ions through the reversible hydration of carbon dioxide^16^. Fifteen members of carbonic anhydrase family are found in humans while the overexpression of CAIX is related to more aggressive neoplasm and inferior therapeutic response^16^. The enzyme contributes to the acidification of the extracellular milieu resulting in poor survival and cancer progression^17^, worse prognosis^18^, and cervical cancers is one of these neoplasms^19^.

Scientists analyzed the importance of CAIX immunostaining for numerous solid tumors, such as renal^20^, vulvar^19^, lung^21^, hepatic^22^, and cervical cancer, both endocervical adenocarcinomas and squamous cell carcinomas^23,24^. In normal tissue, expression of CAIX is limited. Some normal cells, such as gastric, pancreatic duct, crypt cells in small intestine, and gall bladder, express limited amount of CAIX^24^.

In precancerous lesions CAIX tissue immunostaining was described. This was helpful for suspicion of HSIL in atypical squamous cells of undetermined significance (ASCUS) cytology cases^25^, for discrimination of cervical adenocarcinoma in situ from lobular glandular endocervical hyperplasia^26^. CAIX expression is observed in many, but not all cervical intraepithelial neoplasia cases. It is not clear if CAIX positive and CAIX negative CIN cases differ and if any known risk factors or factors under investigation are associated to CAIX expression in the lesion.

The vaginal environment and the presence of carbonic anhydrase IX in cervical tissue has not received as much attention. CAIX expression increases during hypoxic condition aiming to maintain cell pH^27^. Inflammation and hypoxia are intimately linked and contribute to the damage in tumor environment^28^. For example, Ward et al. found that IL-6 expression can increase CAIX expression^29^. CAIX was found to be important for cell motility^16^, better response to radiotherapy in early cervical cancer^30^. CAIX correlated with CD163+ tumor-associated macrophages in cervical cancer^31^. Previous studies confirmed association of CAIX and pro-inflammatory enzyme cyclooxygenase-2 (COX-2) in patients with colorectal cancer^32^. High-risk HPV and CAIX were independent risk factors for head and neck neoplasia^33^. CAIX inhibition promoted apoptosis in cervical cancer cells^34^.

The effective treatment of cervical neoplasia reduced the risk of cervical cancer, but with the cost: the increased probability for negative obstetric outcomes were reported^35^. This notion encouraged other researchers to look for possibilities to stratify patients by evaluating the complexity and interrelation of known risk factors. Bogani et al. developed a normogram, including hr-HPV, extend and severity of disease, age and vaccination status for predicting recurrence of the disease^36^. However, some criteria are finally established after conization. Young age^37^, medroxyprogesterone acetate use among them^38^, *Lactobacilli* spp. dominant microbiome^39^ were related with CIN2 regression and supported a clinical observation tactics. Thus, the identification of more risk factors for disease progression/resolution would be helpful for bedside evidence-based decision making to delay conization after family planning.

Considering importance of smoking, *C. trachomatis* infection, and number of partners, possible association between hr-HPV, CAIX^40^, and microbiota^41^ it is critical to understand, how the expression of CAIX and vaginal microbiota is associated with CIN2+ (CIN2, CIN3 and cervical cancer) in the context of risk factors mentioned above.

The aim of this study was to evaluate the association between vaginal microbiota, including AV and BV, the level of immunohistochemically detected CAIX, and the histological confirmation of CIN2+ in the relation of reported risk factors.

## Materials and methods

### Study design

**Figure 1:**
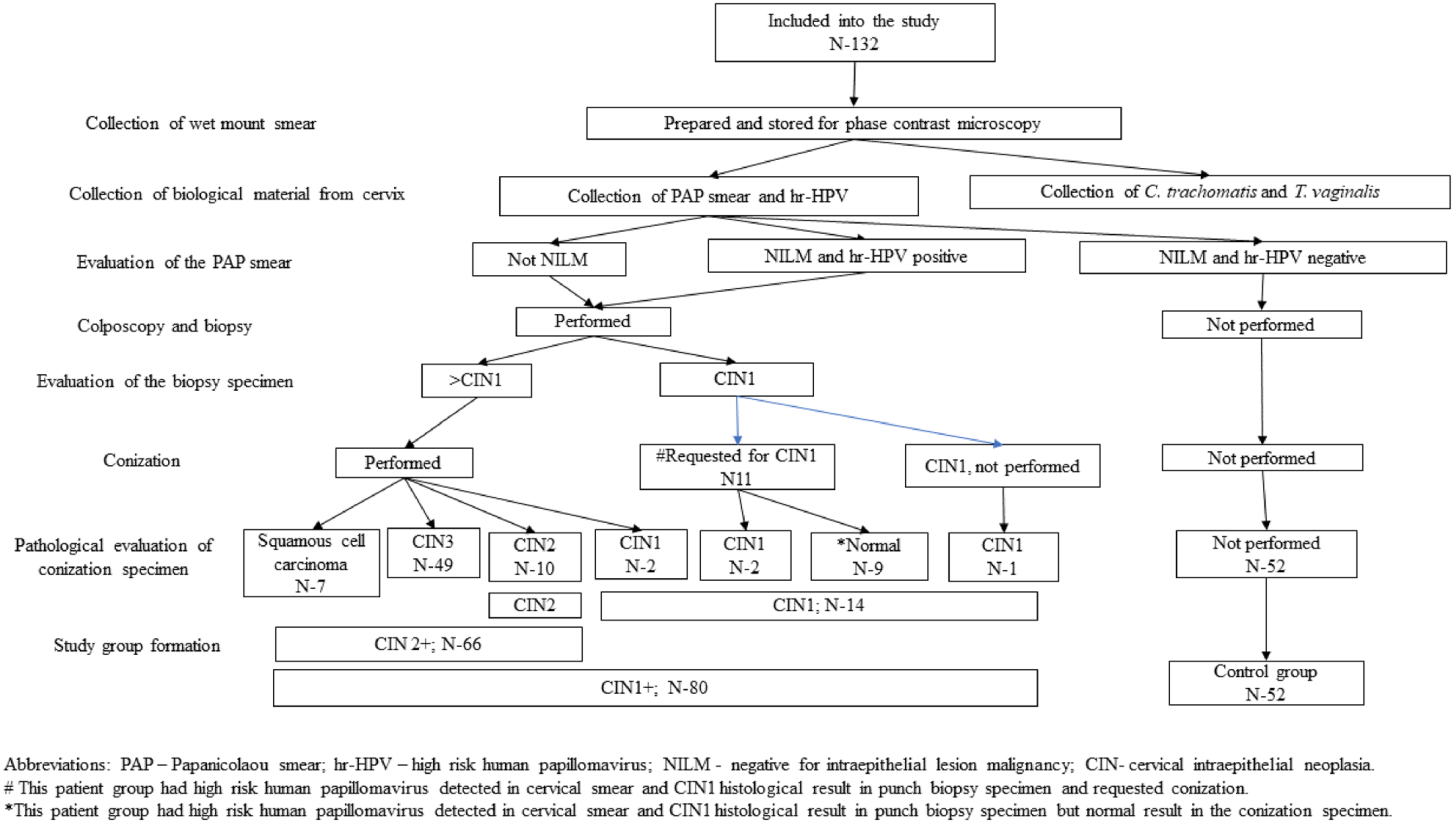
Cervical disease diagnostic workout.

Observational cross-sectional study was conducted^42^ according to the guidelines of the Declaration of Helsinki. The study was approved by the Lithuanian Bioethics committee (permission number 2016-03-22 Nr.: L-16-02/1) and Lithuanian Personal Data Protection Agency. Respondents filled in the questionnaire including their age, education degree, sexual health, and smoking, provided blood, cervical cell and tissue samples as described below. Patients according to education were divided into two groups, women with or without a university-level degree. Flow chart (Fig. 1) provides information on how patients were assigned to the groups. Patient were grouped in the same way as in Plisko 2012^12^ study aiming to increase result comparability for future reviews^43^ as well as for identification of importance of CAIX in the different patient groups.

### Participants

Women (n = 52) with results being negative for intraepithelial lesion or malignancy (NILM) from triennial Papanicolaou test (PAP smear) for National cervical cancer screening program were enrolled into the control group at Onos Gurevičienės Family Clinic in Marijampole city in the same period. Fourteen women with detected hr-HPV and low-grade intraepithelial neoplasia (LSIL) had confirmative colposcopies with punch biopsies at main university affiliated tertial oncogynecology centers (National Cancer Institute and Kaunas Clinics, Lithuanian University of Health Sciences) and were included in the CIN1 group.

Respondents (n = 66; 23–60-year-old) with detected high grade intraepithelial neoplasm on PAP smears, confirmed in colposcopies with punch biopsies were referred for conization to main university affiliated tertial oncogynecology centers (National Cancer Institute and Kaunas Clinics, Lithuanian University of Health Sciences).

Inclusion criteria for both groups were: reproductive age, ability to provide informed written consent, free from hr-HPV and cervical pathology in control group and confirmed cervical pathology and/or hr-HPV in CIN1, CIN2+ group accordingly.

Exclusion criteria were lack of wet mount microscopy data, pregnancy, earlier diagnosed oncological pathology, endometrial hyperplasia, and human immunodeficiency virus (HIV).

### Wet mount smear evaluation

During gynecological exam, unmoistened speculum was used. The vaginal sample was taken with plastic spatula from lower vaginal wall, spread on glass slide through pH strip (range 3.1–7.0; Macherey–Nagel Inc.)^44^. The sample for wet mount microscopy was air dried and transported to the laboratory (Vilnius University Life Sciences Center)^45^. The sample was rehydrated^45^ and evaluated under microscopy. Vaginal pH value was considered normal if it was lower than 4.5.

The microscopic evaluation was performed to assess:

- Microbiota (proportion of lactobacilli (LGB) and background flora, presence of “clue cells”, staphoids or cocci, cytolysis, Candida (pseudohyphae or blastospores).
- Epithelial cells (rate of superficial, parabasal and basal cells).
- Inflammation (count of white blood cells and leucocytes with active vacuoles (so called “toxic”) per high power field/epithelial cell.
- Miscellaneous (erythrocytes, cream, sperm)^46^.

Lactobacilli grade was classified according to Donder’s modified classification described by Schroder^47,48^:

- LGB I—dominance of lactobacilli morphotypes.
- LGB IIa—more than 50% of lactobacilli dominance.
- LGB IIb—less than 50% of lactobacilli detected.
- LBG III—lack of lactobacilli.

Full-blown bacterial vaginosis (BV) was identified if granular microflora dominated and > 20% of clue cells were observed per field. Smears with mixed areas of this type of flora with sporadic “clue cells” and other types of bacteria were classified as partial BV^49^. Aerobic vaginitis (AV) score was calculated following the definition by Donders et al.^14^, considering moderate or severe (msAV) disease ≥ 5.

### Colposcopic evaluation

All patients underwent PAP test and hr-HPV evaluation. After virus detection, colposcopy was performed for CIN detection. Following the standard procedure, the colposcopy-directed biopsies were performed in the cases of cervical abnormalities^50^. After application of 5% acetic acid the transformation zone was evaluated. The visual suspicion of CIN in cervical epithelium were biopsied with forceps.

### Tissue immunohistochemical staining for CAIX

Samples of punch biopsies and conization material were collected into the labeled containers with 10% buffered formalin and sent to the National Center of Pathology, or Department of Pathological Anatomy, Lithuanian University of Health Sciences. Two pathologists (ED and AC) stained the samples with hematoxylin and eosin, evaluated the samples and made a pathological diagnosis as following: CIN1, CIN2, CIN3, CIS (carcinoma in situ), cancer (squamous cell carcinoma), cervicitis, or normal tissue^51^.

Tissue immunohistochemical staining was performed using monoclonal H7 antibodies for CAIX as previously described^52^. Immunohistochemical staining from the conus of the cervical transformation zone was performed using fully automated BenchMark ULTRA IHC/ISH Staining system (Roche, Basel, Switzerland) at the National Center of Pathology and Department of Pathological Anatomy (LUHS). Immunohistochemical staining was performed on both the formalin-fixed and paraffin-embedded tissue samples. EnVision FLEX, High pH kit was used (Dako, Agilent, K8023, Santa Clara, CA, USA) with purified MAb H7^52^ (0.02 mg/ml) as well as hematoxylin (Merck, 75290, Burlington, MA, USA) nuclear staining. Specific immunohistochemical staining was defined by the presence of a brown reaction product on cell membrane under × 40 magnification. Absent membrane staining or faint staining of the cytoplasm was considered negative. Pathologists (ED and AC) confirmed the diagnosis and evaluated immunostaining in atypical cells. They reported staining intensity (negative, weak, moderate, and strong), and the proportion of stained cells following Woelberg^24^. Stained tissues were photographed using ScanScope XT system (Aperio, Leica Biosystems Inc., Bualo Grove, IL, USA).

### hr-HPV DNA detection by genotyping

During first visit biological material from the cervix was obtained with cervical brush (Rovers medical devices, Oss, Netherlands) into the “CyMol” medium (Copan, Brescia, Italy) for transportation. Clinical samples were centrifuged at 2000×*g* for 15 min at 4 °C and hr-HPV DNA was extracted using GeneJet Genomic DNA Purification Kit (Thermo Fisher Scientific, Vilnius, Lithuania) according to the manufacturer’s protocol. Genomic DNA was eluted with 100 μl of Elution Buffer. The purified DNA was used for PCR analysis or stored at − 20 °C^53^.

### Chlamydia trachomatis detection

Clinical samples from cervix were using a brush and transformed to a vial of liquid preservative for the determination of *C. trachomatis*. The DNA extraction was performed using the GeneJET Genomic DNA Purification Kit (Thermo Fisher Scientific, Lithuania)^54^.

### Statistical analysis

Data were analyzed using SPSS 28 software. Results were presented in proportions, means, medians and ranges. Mann–Whitney U test (MWU) was used for comparison of independent samples. Asymptotic exact sign or Fisher’s exact test p value was interpreted as statistically significant if p < 0.05. The risk of CIN2+ development depending on various risk factors was calculated as odds ratios.

### Ethics approval and consent to participate

The study was conducted according to the guidelines of the Declaration of Helsinki. The study was approved by the Lithuanian Bioethics committee: L-16-02/1 and Lithuanian Personal Data Protection Agency.

## Results

For the comparative analysis, 52 women were in the control group and 80 in the CIN1+ group (CIN1, CIN2, CIN3 and cancer). In the CIN1+ group, the histological evidence of cervicitis was diagnosed for three patients, and they were moved to the control (no CIN) group. Eight respondents in each group were excluded due to unreadable microscopy slides. Participants’ age varied between 23 and 60 years old. Healthy women were slightly older (p < 0.01) and most of them—cigarette non-users (p < 0.00). Smoking was related to earlier coitarche (17 ± 1.6 vs 18 ± 2.6, MWU p < 0.01), younger age (35.4 ± 5.8 vs 40.2 ± 9.9; MWU p = 0.01), and the number of partners (3.1 ± 1.7 vs 2.2 ± 1.3; MWU p = 0.02). The difference in education level was insignificant between the groups. Higher proportion of less educated women with cervical pathology smoked (27/35 77.1% vs 15/43 34.9%; p = 0.00), most of them were diagnosed with CIN2+ (n = 21). The tendency was not observed in the control (no CIN) group (Table 1).

**Table 1:** Characteristics of study group participants with CIN1 + and controls (no CIN).

Characteristics	No CIN	CIN1 +	p value
	N = 52	N = 80	
Mean age	41.6 ± 9.7	36.2 ± 7.8	0.01^$^
Mean menarche age	13.6 ± 1.7	13.7 ± 1.6	0.38^$^
Mean coitarche age	18.7 ± 2.5	18.1 ± 2.3	0.07^$^
Mean number of partners	1.9 ± 0.9	3.1 ± 1.6	0.00^$^
Mean number of pregnancies	2.3 ± 1.4	1.9 ± 1.8	0.03^$^
Mean number of abortions	0.6 ± 0.8	0.6 ± 1.0	0.51^$^
Mean number of deliveries	1.7 ± 0.8	1.5 ± 1.1	0.06^$^
Education level**			
Higher	34 (68.0%)	44 (55.0%)	0.41
Other*	16 (32.0%)	36 (45.0%)	
Smoking***			
Yes	8 (15.4%)	42 (53.8%)	0.00
No	44 (84.6%)	36 (46.2%)	
Contraception method			
Male condom	7 (13.5%)	21 (26.3%)	0.08
Intrauterine device	5 (9.6%)	2 (2.5%)	0.08
Intrauterine hormonal system	2 (3.8%)	3 (3.7%)	1.0
Oral contraceptives	7 (13.5%)	6 (7.5%)	0.26
None	27 (51.9%)	45 (56.3%)	0.63
Other	4 (7.7%)	3 (3.7%)	1.0
Sexually transmitted disease detection			
Chlamydia trachomatis positive	2 (18.2%)	7 (29.2%)	0.68
Trichomonas vaginalis positive	0 (0%)	1 (1.3%)	0.30

*Primary, secondary or vocational education.

**Two people did not report their education, thus the number of participants in “No CIN” group was 50 and the total was 130.

***Two persons did not report their smoking status, thus the number of participants in CIN1+ group was 78 and the total was 130.

^$^MWU p value.

The histological findings in study group followed as CIN1 (n = 14), CIN2 (n = 10), CIN3 (n = 49), and cancer (n = 7; all were cervical squamous cell carcinomas). In the cases when several grades of lesion were detected, the most advantageous lesion was included in the analysis.

Table 2 shows the differences in pH value and microbiota findings among respondents with and without intraepithelial lesion. Lactobacilli grade variation was insignificant between the groups. Vaginal pH that increases in the cases of flora alteration was higher in CIN1+ or CIN2+ that in controls. Women with cervical pathology had more leucocytes, and msAV. Prevalence of bacterial vaginosis (both any (p > 0.05) or just full blow vaginosis (p > 0.05) did not differ between the groups. The main reason was the higher prevalence of msAV among CIN2+ (53.0%) than CIN1 (21.4%) (Table 2). BV cases between the groups variated insignificantly both for any (p > 0.05) or just full blow vaginosis (p > 0.05).

**Table 2:** Wet mount microscopy findings and pH in accordance with CIN severity groups.

	No CIN	CIN1	CIN1+	CIN2+	No CIN vs CIN1+	CIN1 vs CIN2+	No CIN vs CIN2+
	N = 52	N = 14	N = 80	N = 66	*p* value		
Vaginal pH							
pH < 4.5	30 (57.7%)	7 (50.0%)	20 (25.0%)	13 (19.7%)	0.00	0.17	0.00
pH ≥ 4.5	22 (42.3%)	7 (50.0%)	60 (75.0%)	53 (80.3%)			
Lactobacilliary grade							
LGB I	18 (34.6%)	4 (28.6%)	17 (21.3%)	13 (19.7%)	0.39	0.91	0.32
LGB IIa	7 (13.5%)	2 (14.3%)	12 (15.0%)	10 (15.2%)			
LGB IIb	6 (11.5%)	2 (14.3%)	13 (16.3%)	11 (16.7%)			
LGB III	21 (40.4%)	6 (42.9%)	38 (47.5%)	32 (48.5%)			
Vaginal leucocytosis							
< 10 hpf	42 (80.8%)	10 (71.4%)	36 (45.0%)	26 (39.4%)	0.001	0.16	0.001
< 10/epithelial cell cell	4 (7.7%)	2 (14.3%)	22 (27.5%)	20 (30.3%)			
> 10/epithelial cell	3 (5.8%)	2 (14.3%)	13 (16.3%)	11 (16.7%)			
Full hpf	3 (5.8%)	0 (0%)	9 (11.3%)	9 (13.6%)			
Presentation of bacterial vaginosis (BV)							
No BV	32 (61.5)	9 (64.3)	60 (75.0)	51 (77.3)	0.10	0.31	0.06
Any BV	20 (38.5)	5 (35.7)	20 (25.0)	15 (22.7)			
Presentation of aerobic vaginitis (AV)							
No AV	43 (82.7)	11 (78.6)	42 (52.5)	31 (47.0)	0.00	0.04	0.00
msAV	9 (17.3)	3 (21.4)	38 (47.5)	35 (53.0)			
CAIX immunostaining							
No/weak	3 (100)	8 (100)	53 (62.3)	45 (69.2)	0.56	0.09	0.55
msCAIX	0 (0)	0 (0)	20 (37.7)	20 (30.8)			

Numbers in % are shown in the brackets.

*CIN* cervical intraepithelial neoplasia, *LGB* lactobacilli grade, *hpf* high power field, *BV* bacterial vaginosis, *AV* aerobic vaginitis, *msAV* moderate/severe aerobic vaginitis, *msCAIX* moderate/severe carbonic anhydrase immunostaining.

**Figure 2:**
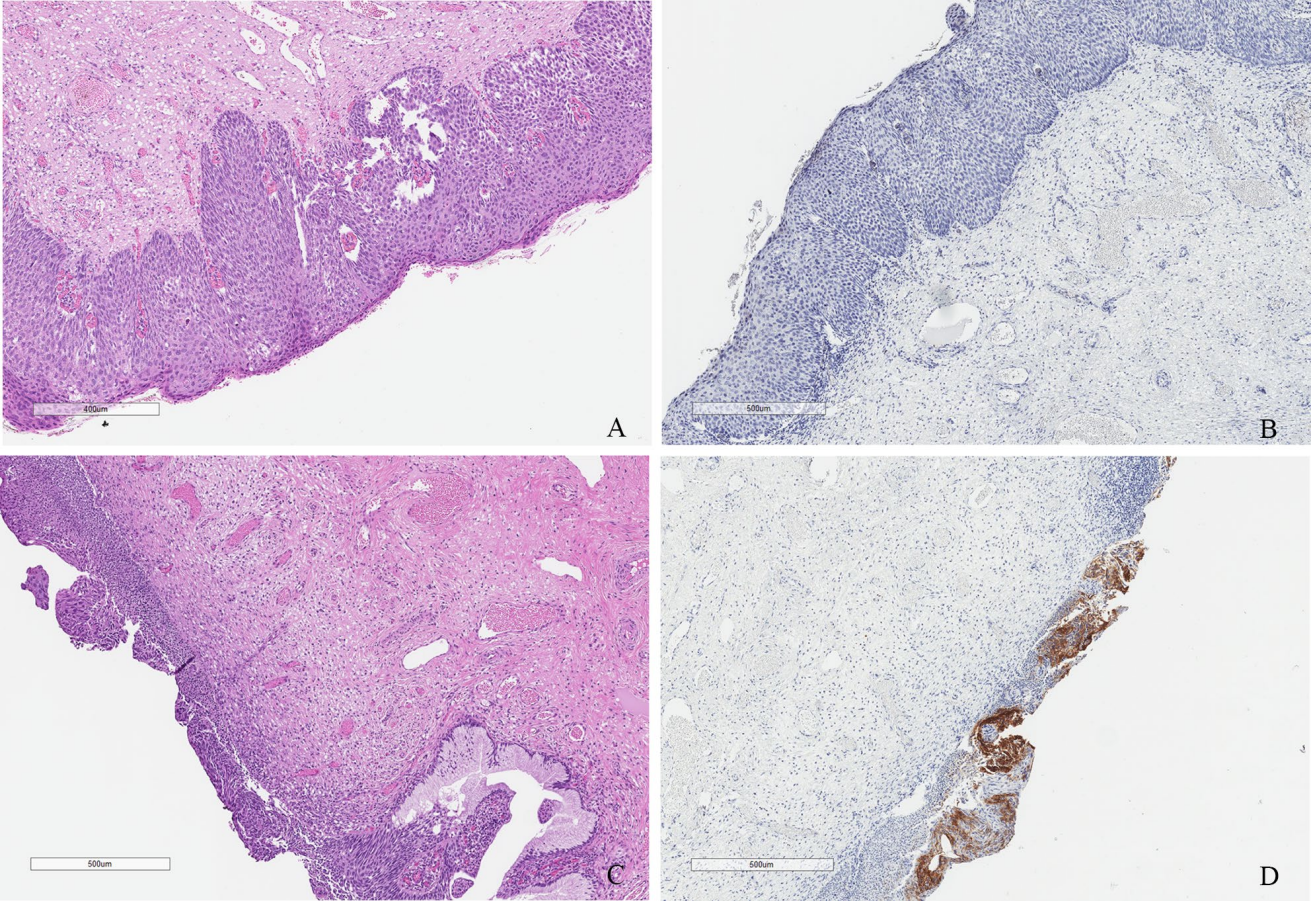
Immunohistochemical staining with CAIX on surgical specimens. The first sample was immunohistochemically stained with hematoxylin and eosin (**A**) and CAIX with negative results (**B**). The second sample was immunohistochemically stained with hematoxylin and eosin (**C**) and CAIX with positive results (**D**).

The immunohistochemical staining with CAIX antibodies was performed on surgical specimens (Fig. 2). The reaction was negative or weak for three cases without dysplasia, 8 of LSIL, 53 CIN1+ and 45 CIN2+. Moderate or strong immunohistochemical staining was observed among CIN2+ only in 20 cases. However, co-existence of msAV and msCAIX showed different pattern.

In CIN2+ group msAV was more prevalent in non-stained group than in msCAIX (29; 82.8% vs 6; 17.2% respectively, p = 0.02). Subgroup analysis revealed the msCAIX staining without msAV (N = 4) or msAV without CAIX staining (N = 5) (p = 0.048) only in CIN2 only group. None had both msAV and msCAIX immunohistochemical staining in that subgroup. However, in CIN3 only group this tendency was statistically insignificant—patients had both msAV and msCAIX in six CIN3 cases (p = 0.37). The prevalence of BV was not related to msCAIX staining (p = 1.0).

Higher number of partners and smoking were more prevalent in women with CIN2+ (OR 0.94 (95% CI 0.89–0.98), p = 0.01 and OR 7.1 (95% CI 2.7–17.4), p < 0.01) compared to women without cervical dysplasia. Microbiota alteration such as msAV (OR 5.3 (95% CI 2.3–12.8), p < 0.01) was more frequent in study group than controls (Table 3).

**Table 3:** Result of univariate and multivariate regression between the risk factors such as smoking, number of partners, and moderate or severe aerobic vaginitis (msAV) and CIN2+ condition.

	Univariate logistic regression			Multivariate logistic regression		
	Odds ratio	95% CI	p value	Odds ratio	95% CI	p value
Smoking	7.1	2.9–17.4	< 0.01	5.4	2.0–14.7	< 0.01
Number of partners	2.0	1.4–2.7	0.01	1.7	1.2–2.5	0.01
msAV	5.4	2.3–12.8	< 0.01	5.7	2.1–15.2	< 0.01

*msAV* moderate/severe aerobic vaginitis.

Multivariate logistic regression covered analysis of number of partners, smoking and msAV. All of them were independent risk factors for CIN2+ increasing the risk of the disease five times if msAV is diagnosed or smoking is present.

## Discussion

This study analyzed the association between vaginal dysbiosis, grade of cervical intraepithelial lesion, and CAIX expression. The moderate or severe dysplasia is usually detected in the mild dysplasia area due to progression^55^, so we considered the most advanced grade of lesion trying to understand co-factors of the process. Results showed that microbiota alteration such as msAV was associated with higher grade intraepithelial lesions (CIN2+), when both smoking and number of partners were addressed. Moreover, msAV was detected together with msCAIX only in more advanced (CIN3) cases. This was not a case among patients with CIN2.

The association between intraepithelial lesion and behavioral risk factors such as smoking or the number of partners have been described in other studies^56–59^ as well as the relation of elevated vaginal pH, abnormal vaginal microbiota diagnosed on wet mount microscopy and cervical precancerous lesions^13,60,61^. Our results were in line with these findings.

Studies show that hr-HPV carcinogenesis is associated with local inflammation^62^, increased number of leucocytes in vaginal fluid and concentration of inflammatory interleukins (IL)^63,64^. Inflammatory microenvironment is genotoxic, stimulates epigenetic changes and DNA damage in the cervix.

*Lactobacilli* spp. increase adenosine and cytosine levels and reduce inflammation^65^, but they are suppressed in msAV and BV cases. Vaginal microbiome called community state type IV with decreased number of *Lactobacilli* spp. was associated with CIN2+ persistence after 24 months^39^. However, in this study, community state type IV was not differentiated into subtypes I and II in accordance with prevalence of anaerobic (in BV cases) or aerobic (in AV cases) bacteria. Despite lack of leucocytes, modern metabolomic analysis shows that BV maybe also related to proinflammatory dysbiosis with elevated cervicovaginal cytokines and chemokines, such as IL-1β, IL-6, and IL-8^66,67^.

We did not find differences of lactobacilliary grade between control group and patients with cervical anomaly as well as between low and high grade cervical intraepithelial neoplasia. After comparison of two types of dysbiosis (BV and AV), the proportion of respondents with bacterial vaginosis was similar in all groups. Plisko et al. results were similar—AV, but not BV was related to cervical dysplasia^12^. Plisko et al. authors hypothesized that the inflammatory characteristics of msAV and hr-HPV-induced cervix dysplastic lesions, are crucial for the progression of lesions towards invasive cancer^12^.

Moreover, highly increased concentrations of IL-1-β and IL-6, that promoted CAIX expression^29^, were observed in msAV^15^. Enterococcus (part of msAV flora) was associated with elevation of IL-6 and IL-8 also in Moscicki et al. study^41^. Escherichia coli (that is also found in msAV) produced the 2-hydroxyglutarate (oncometabolite) in colorectal carcinogenesis^68^.

Plisco et al. discussed that BV can be seen as an indirect marker of sexual behavior leading to hr-HPV acquisition rather than be directly involved in the pathogenesis of cervical cancer^12^. Another study found that multiple sexual partners were associated with hr-HPV DNR detection^69^, that could contribute to higher CIN1 rate.

We found that BV was not related to msCAIX immunostaining. The msAV was not prevalent in msCAIX stained CIN2 group but was found among patients with CIN3. Thus, we speculate that presence of msAV and msCAIX staining could be associated with CIN2 progression to CIN3. However, we do not know if microbiota alteration is directly related to increased hypoxia or other mechanisms are affecting inflammatory and hypoxia process during progression of the lesion.

The cross-sectional study design was sufficient to find an association but is not sufficient for observation of the lesion progression. Retrospective data about sexual habits and risk factors usually limits the possibility to identify association with intensity of exposition. However, this was not our aim. Microbiota was evaluated, but microbiome and metabolomic analysis wasn’t performed and could be seen as limitation of the study as well as a possible direction for further research.

The strength of the study was the use of standard procedures for colposcopy and wet mount microscopy, confirmation of severity of cervical lesions by histologic evaluation of biopsies before treatment and in conuses after surgery, as well as automatized standardized CAIX immunostaining. Thus, this enables comparability between the studies for further meta-analysis.

More studies are needed to understand the relation of abnormal vaginal microbiome and the development of cervical cancer and more aggressive disease in hr-HPV-positive women. Further evaluation of importance of msCAIX and msAV interplay and interrelation with behavioral factors is necessary for drawing the whole picture of interrelations among risk factors. It is also important to clarify whether the treatment of abnormal vaginal microbiota could help prevent the development of cervix cancer or could contribute to cancer prognosis.

## Conclusions

The alteration of vaginal microbiota, such as msAV, is associated with more advanced cervical intraepithelial lesions even if behavioral risk factors, such as smoking, and the number of partners is considered. The associated inflammation due to msAV and increased CAIX expression in cervical cells should be taken into consideration when studying vaginal microbiota in women with pre-invasive cervical lesions.

## Data Availability

Due to the risk that individual privacy could be compromised, supplemental data and material are not available publicly**.** The availability of data was inspected by Lithuanian Personal Data Protection Agency. If someone wants to request the data from this study they should contact svitrigaile.grinceviciene@bti.vu.lt.

## Abbreviations

CAIX: Carbonic anhydrase IX
CIN: Cervical intraepithelial neoplasia
BV: Bacterial vaginosis
msAV: Moderate or severe aerobic vaginitis
hr-HPV: High-risk human papillomavirus
DNA: Deoxyribonucleic acid
HSIL: High-grade intraepithelial neoplasia
LSIL: Low-grade intraepithelial neoplasia
AV: Aerobic vaginitis
NILM: Intraepithelial lesion or malignancy
PAP: Papanicolaou test
MWU: Mann–Whitney U test
